# Bone Regeneration Potential of Human Dental Pulp Stem Cells Derived from Elderly Patients and Osteo-Induced by a Helioxanthin Derivative

**DOI:** 10.3390/ijms21207731

**Published:** 2020-10-19

**Authors:** Marika Sato, Yoko Kawase-Koga, Daiki Yamakawa, Yasuyuki Fujii, Daichi Chikazu

**Affiliations:** 1 Department of Oral and Maxillofacial Surgery, Tokyo Medical University, 6-7-1 Nishishinjuku, Shinjuku-ku, Tokyo 160-0023, Japan; d06048@yahoo.co.jp (M.S.); daiki19@tokyo-med.ac.jp (D.Y.); yasuyuki.fujii0730@gmail.com (Y.F.); chikazu@tokyo-med.ac.jp (D.C.); 2 Department of Oral and Maxillofacial Surgery, School of Medicine, Tokyo Women’s Medical University, 8-1 Kawada-cho, Shinjuku-ku, Tokyo 160-0023, Japan

**Keywords:** dental pulp stem cells, cell sheet, bone regeneration, age, osteogenic differentiation

## Abstract

Human dental pulp stem cells (DPSCs) have high clonogenic and proliferative potential. We previously reported that a helioxanthin derivative (4-(4-methoxyphenyl)pyrido[40,30:4,5]thieno[2–b]pyridine-2-carboxamide (TH)) enhances osteogenic differentiation of DPSCs derived from young patients. However, in the clinical field, elderly patients more frequently require bone regenerative therapy than young patients. In this study, we examined and compared the osteogenic differentiation potential of TH-induced DPSCs from elderly patients and young patients to explore the potential clinical use of DPSCs for elderly patients. DPSCs were obtained from young and elderly patients and cultured in osteogenic medium with or without TH. We assessed the characteristics and osteogenic differentiation by means of specific staining and gene expression analyses. Moreover, DPSC sheets were transplanted into mouse calvarial defects to investigate osteogenesis of TH-induced DPSCs by performing micro-computed tomography (micro-CT). We demonstrated that osteogenic conditions with TH enhance the osteogenic differentiation marker of DPSCs from elderly patients as well as young patients in vitro. In vivo examination showed increased osteogenesis of DPSCs treated with TH from both elderly patients and young patients. Our results suggest that the osteogenic differentiation potential of DPSCs from elderly patients is as high as that of DPSCs from young patients. Moreover, TH-induced DPSCs showed increased osteogenic differentiation potential, and are thus a potentially useful cell source for bone regenerative therapy for elderly patients.

## 1. Introduction

Bone regenerative therapy is in great demand nowadays for alveolar bone loss caused by periodontitis, tumor resection, traumatic injury, and dental implant therapy. In the clinical field, bone regenerative therapy is required especially for elderly patients over 40 years of age, owing to the high morbidity rate of periodontitis. Mesenchymal stem cells (MSCs) derived from bone marrow (BMSCs) and adipose tissue are known to be a useful cell source for tissue engineering. However, collecting BMSCs is challenging, owing to the necessity of using an invasive technique. To date, bone augmentation techniques such as bone harvesting from the iliac crest under general anesthesia, autogenous bone grafting from the mandible, and artificial bone grafting have been used for alveolar bone defect in periodontitis treatment and dental implant treatment. However, these present a high likelihood of infection and unexpected resorption.

Human dental pulp stem cells (DPSCs) were identified in 2000, and they have a high potential to proliferate, self-renew, and differentiate into multiple lineage cells such as adipogenic, neurogenic, and osteogenic cells [1,2,3]. The clonogenic and proliferative potential of DPSCs is higher than that of BMSCs [4]. Moreover, DPSCs can be obtained from extracted teeth that are usually discarded, and the method of collecting DPSCs is non-invasive and uncomplicated without ethical issues. Thus, DPSCs have the potential to be a promising cell source for regenerative therapy [1,2,5].

Several studies report that the number of MSCs and stem cell functions such as the ability to self-renew decline with increasing age [6]. Furthermore, the proliferation and differentiation potential of MSCs also declines with age and after repeated passage [7,8,9,10], owing to telomere shortening, DNA damage, and epigenetic changes in transcriptional regulation [11]. Although many studies have investigated the differentiation potential of DPSCs isolated from young patients [4,12,13], little information is available about osteogenic differentiation potential of DPSCs derived from elderly patients. In fact, the volume of dental pulp tissue decreases with age, owing to secondary dentin formation and root canal mineralization [14]. Thus, a simple and effective method of transplantation is required to overcome these limitations.

Recently, cell sheet technology has been widely studied not only in bone and cartilage but also in cornea and heart regenerative therapy [15,16]. Cell sheet technology facilitates stem cell harvest and transplantation without the need for digestive enzymes and scaffolds, resulting in minimal cell loss and damage and avoidance of inflammatory responses caused by biodegradable scaffolds [17]. Thus, cell sheet technology has attracted attention as a cell transplantation system that could overcome the shortcoming of conventional tissue engineering technology. In our previous study, we showed that 4-(4-methoxyphenyl)pyrido[40,30:4,5]thieno[2–b]pyridine-2-carboxamide (TH)—a small-molecule derivative of helioxanthin—induces osteogenic differentiation of DPSCs isolated from young patients in vitro and in vivo, using transplantation of DPSCs with cell sheet technology [18]. Although the mechanism underlying TH on promoting osteogenic differentiation is still largely unknown, a recent study showed that TH inhibited the activity of phosphodiesterase (PDE) on cyclic guanosine monophosphate (cGMP) by stimulating nitric oxide (NO) production, indicating TH might promote bone formation partially through PDE inhibition [19]. However, the effect of TH on the osteogenic differentiation potential of DPSCs derived from elderly patients remains unknown. We hypothesized that TH can promote osteogenic differentiation and osteogenesis of DPSCs from elderly patients as well as young patients.

In the present study, we investigated and compared the osteogenic differentiation potential and osteogenesis of TH-induced DPSCs from elderly and young patients in vitro and in vivo to explore the potential clinical application of DPSCs.

## 2. Results

### 2.1. Characterization of Young and Elderly Dental Pulp Stem Cells

**Figure 1:**
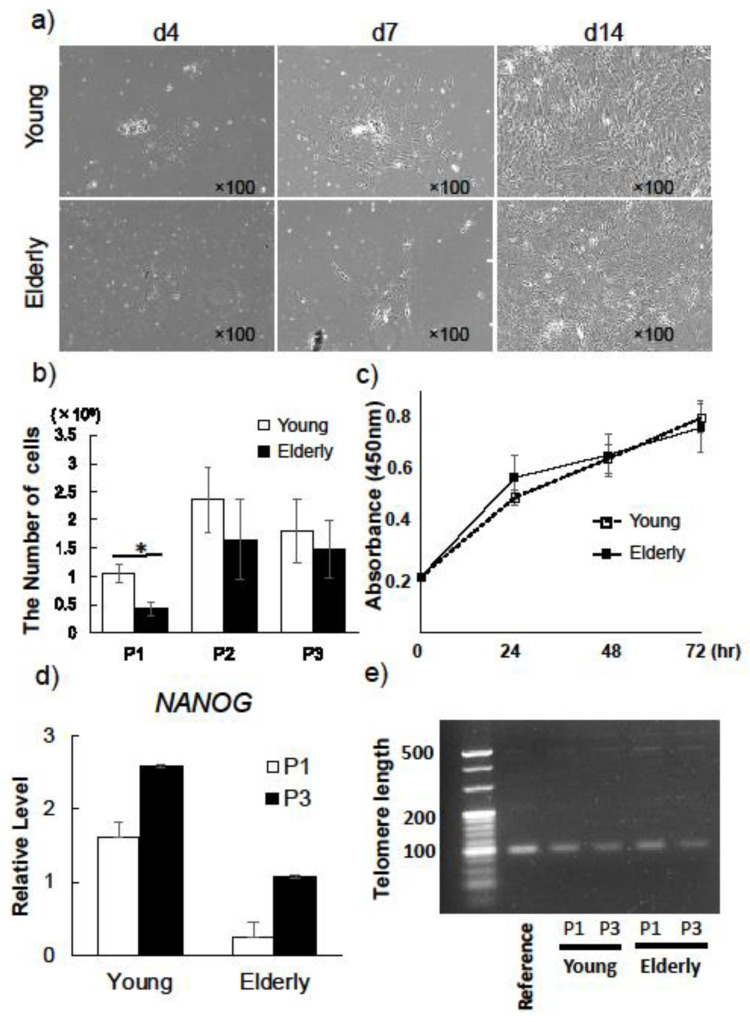
Characterization of dental pulp stem cells (DPSCs) derived from young and elderly patients. (**a**) Cell morphology of DPSCs derived from young and elderly patients at initial culture. Gross view at day 4, day 7, and day 14. (**b**) Comparison of proliferation potential of DPSCs derived from young and elderly patients in passage 1 (P1) to P3. The number of collected cells was counted at each passage (*n* = 4) (* *p* < 0.05). (**c**) Proliferation ability of young and elderly DPSCs at P2 were analyzed at 24, 48, and 72 h of culture. (*n* = 4) (* *p* < 0.05). (**d**) Comparison of expression of stem cell marker (NANOG) in the DPSCs derived from young and elderly patients at P1 and P3 (*n* = 4) (* *p* < 0.05). (**e**) Comparison of telomere length in the DPSCs derived from young and elderly patients at P1 and P3. The reference genomic DNA sample with known telomere length is shown on the left (*n* = 3). The experiments were repeated three times, and one representative image is presented.

Young and elderly DPSCs showed morphologically similar spindle shape at initial culture (Figure 1a). The average number of DPSCs derived from young and elderly patients was 70% and 42% of the total number of cells collected from dental pulp, respectively (Table 1). Proliferation potential of elderly DPSCs in passage 1 (P1) was significantly lower than that of young DPSCs (Figure 1b). However, in P2, the proliferation potential of elderly DPSCs increased and was not significantly different from that of young DPSCs—it remained at a high level in P3 (Figure 1b). Moreover, we used colorimetric assay to analyze the proliferation ability at 24, 48, and 72 h of culture in P2 (Figure 1c). There were no significant differences in proliferation rates between young and elderly DPSCs at each time. Thus far, it has been suggested that elderly DPSCs after P2 had proliferation ability as high as young DPSCs. 

We next assessed and compared the expression of the stem cell marker NANOG and telomere length between young and elderly DPSCs at P1 and P3. The expression level of NANOG by real-time PCR showed a similar pattern between P1 and P3 in both young and elderly DPSCs (Figure 1d). The telomere length, known as a marker of cellular senescence, remained stable in young and elderly DPSCs from P1 to P3 (Figure 1e). These results suggested that the stem cell property remained stable in both young and elderly DPSCs until at least P3.

**Figure 2:**
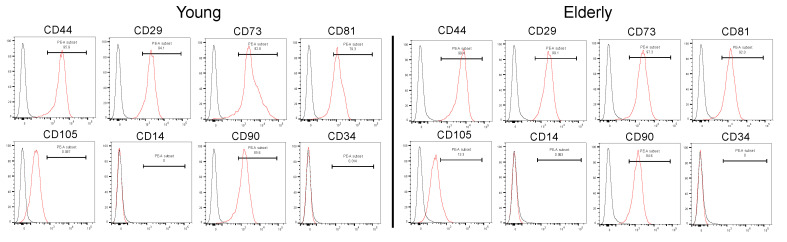
Flow cytometry analysis of dental pulp stem cells (DPSCs) derived from young and elderly patients. DPSCs were cultured at passage 3 (P3) or P4 and surface stained with phycoerythrin-conjugated CD14, CD29, CD34, CD44, CD73 CD81, CD90, and CD105. Fluorescent signals were measured using flow cytometry analysis. Cells that were not treated with fluorescent antibodies were used as controls. Isotype controls are represented by black curves, and labelled cells are represented by red curves. The experiments were repeated three times (*n* = 4), and one piece of representative data is presented.

Flow cytometry analysis indicated that young and elderly DPSCs showed a similar pattern of expression of MSC surface markers, being positive for CD29, CD44, CD73, CD81, CD90, and CD105, and negative for CD14 and CD34 (Figure 2), which are the minimal criteria for DPSCs.

### 2.2. Effect of TH on Osteogenic Differentiation of Young and Elderly Dental Pulp Stem Cells

**Figure 3:**
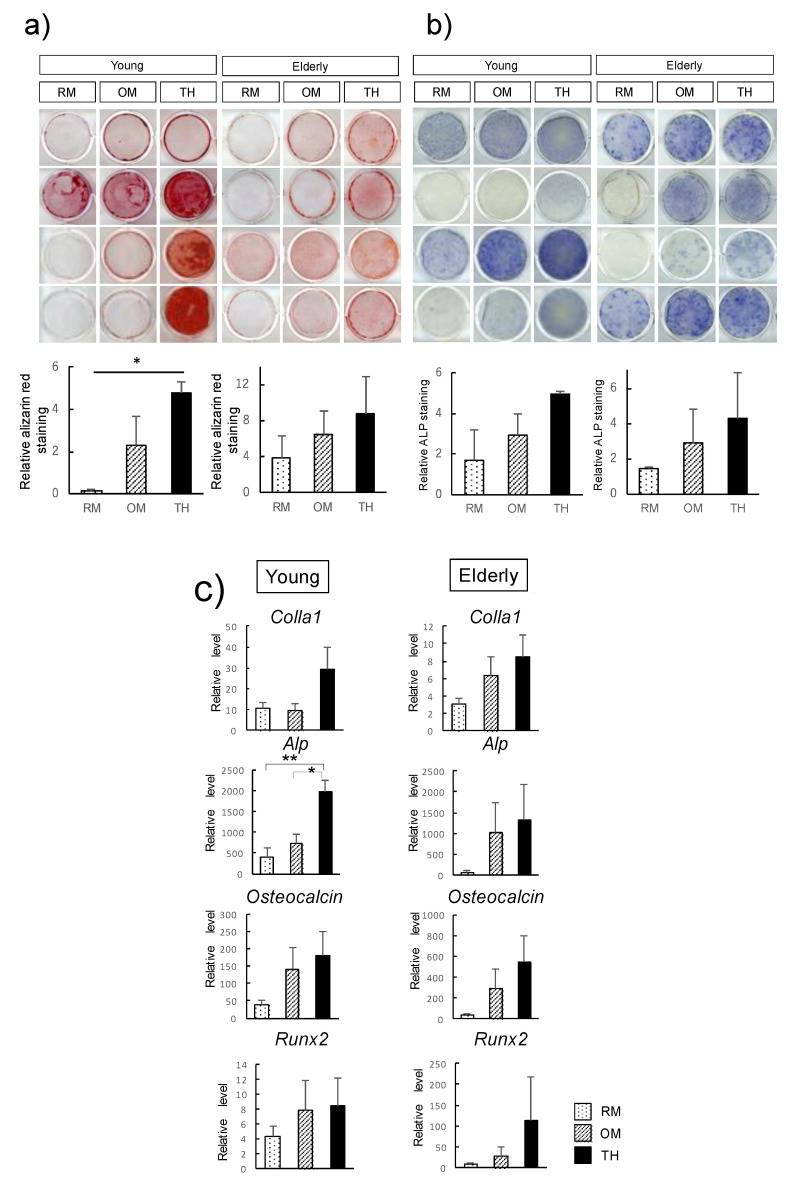
Effect of 4-(4-methoxyphenyl)pyrido[40,30:4,5]thieno[2–b]pyridine-2-carboxamide (TH) on the osteogenic differentiation of dental pulp stem cells (DPSCs) derived from young and elderly patients. (**a**) Young and elderly DPSCs were cultured in regular medium (RM), osteogenic medium (OM), or OM with TH for 14 days. Cells were stained with alizarin red S to detect matrix mineralization (*n* = 4). Quantification of alizarin red S staining was calculated in arbitrary units. (**b**) To detect alkaline phosphatase (ALP) activity, we subjected cells to ALP staining (*n* = 4). Quantification of ALP staining was calculated in arbitrary units. (**c**) Real-time PCR was performed to determine the expression levels of osteogenic differentiation markers in young and elderly DPSCs cultured in RM, OM, or OM with TH for 14 days (*n* = 4). Error bars represent the standard deviation. Statistical analyses were performed using one-way ANOVA (* *p* < 0.05, ** *p* < 0.01). The experiments were repeated three times.

To determine the effect of TH on elderly DPSCs, we compared the osteogenic differentiation potential of TH-induced DPSCs from young and elderly patients. Alizarin red S staining showed that there was higher calcification in DPSCs cultured in osteogenic medium (OM) with TH than in OM without TH in both young and elderly DPSCs (Figure 3a). Moreover, alkaline phosphatase (ALP) staining showed that young and elderly TH-induced DPSCs displayed stronger ALP activity than DPSCs in OM (Figure 3b). Real-time PCR showed that TH upregulated osteogenic differentiation markers in DPSCs derived from both young and elderly patients, although the difference was not significant. In addition, in DPSCs from both young and elderly patients, we found ColIa1, Alp, osteocalcin, and Runx2 to be more highly expressed in TH-induced DPSCs than in DPSCs cultured in OM, although the difference was not statistically significant (Figure 3c). 

**Figure 4:**
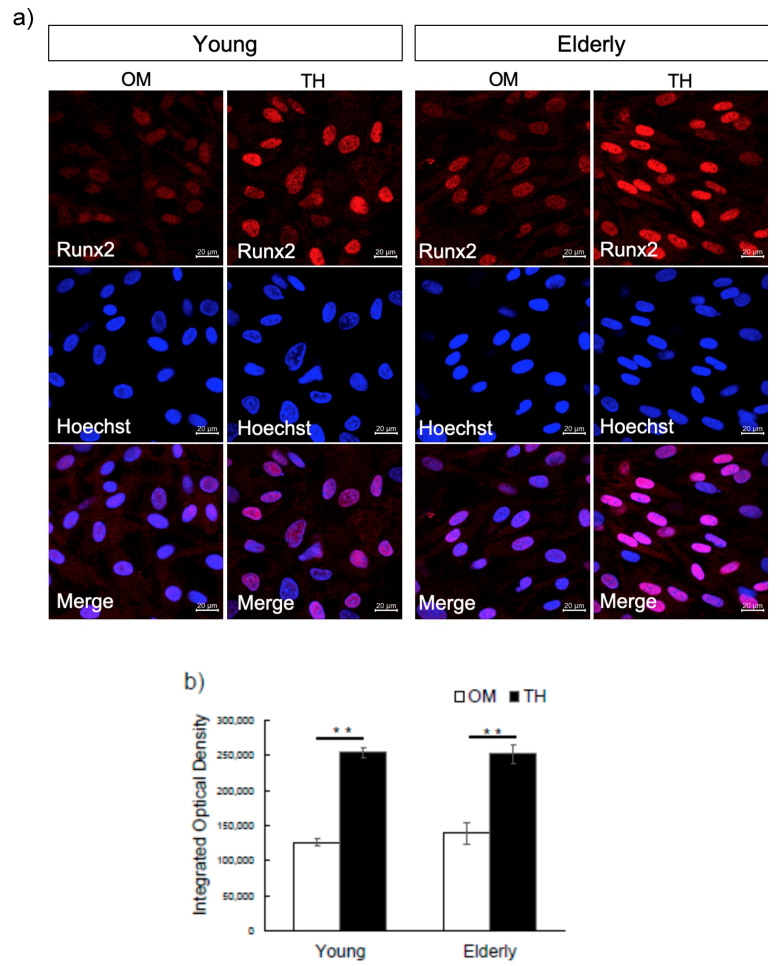
The effect of TH on Runx2 expression of DPSCs from young and elderly patients. (**a**) Young and elderly DPSCs were cultured in OM and OM with TH for 7 days. The cells were stained by antibodies of Runx2. Nuclei were visualized by Hoechst. Original magnification was ×40. Scale bar is 20 µm. (**b**) Integrated density of nuclei of 30 independent cells in randomly selected field of view was measured. Analysis of fluorescent signal of nuclei of cells treated with TH and OM of three independent experiments was performed. Statistical analysis was performed by Student’s *t*-test. (** *p* < 0.01).

To gain further insight into the effect of TH on osteogenic differentiation, we next detected the expression of Runx2 by performing immunocytochemical analysis. The immunocytochemistry revealed that expression of Runx2 was stronger in both young and elderly TH-induced DPSCs compared to that in OM (Figure 4a). Next, we compared the nuclear intensity of Runx2 immunofluorescent signals detected in TH-induced DPSCs with that in OM. TH upregulated integrated density levels of nuclear Runx2 in comparison with OM at a significant level in both young and elderly DPSCs (Figure 4b). 

### 2.3. In Vivo Osteogenesis of TH-Induced Young and Elderly Dental Pulp Stem Cells

**Figure 5:**
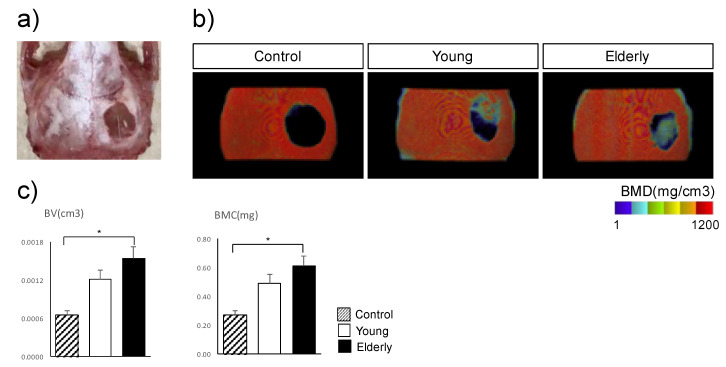
Radiological findings of mouse calvarial defects 8 weeks after transplantation with cell sheets of TH-induced dental pulp stem cells (DPSCs) from young and elderly patients. (**a**) TH-induced DPSC sheets were cultured on temperature-responsive dishes for 14 days and then transplanted into mouse calvarial defects (3.5 mm in diameter). Eight weeks after transplantation, we dissected the calvaria (*n* = 4). (**b**) Micro-computed tomography images indicated the bone mineral density (BMD) values. The experiments were repeated three times, and one representative image is shown. (**c**) Quantification of bone volume (BV) and bone mineral content (BMC) of a 5 × 5 × 3 mm^3^ cuboid area in the center of a circular defect. Data are expressed as means ± standard deviation of five mice per group. * *p* < 0.05 by Student’s unpaired two-tailed *t*-test.

To determine the osteogenesis of TH-induced elderly DPSCs in vivo, we cultured young and elderly DPSCs on temperature-responsive dishes (12-well, 3.5 cm^2^/well) under osteogenic conditions with TH for 2 weeks, then harvested the DPSC sheets and transplanted them into the calvarial defects of mice. Eight weeks after transplantation, micro-computed tomography (micro-CT) images were taken to compare the bone regeneration potential of young DPSCS with that of elderly DPSCS (Figure 5a). The micro-CT images revealed that the newly formed bone was detected at the edge of the defect in the elderly DPSC group as well as in the young DPSC group, whereas no bone regeneration was observed in the control group (Figure 5b). Quantitative evaluation indicated no significant differences in bone volume (BV) and bone mineral content (BMC) between young and elderly DPSC groups; however, BV and BMC in the elderly DPSC group were significantly higher than those in the control group (Figure 5c).

## 3. Discussion

In the present study, we studied the osteogenic differentiation potential of TH-induced DPSCs from elderly patients and compared it with that of DPSCs from young patients. In comparison with BMSCs [2] and adipose tissue-derived stem cells (ADSCs) [20], DPSCs have a higher proliferative potential and ease of accessibility, and the method to harvest them is less invasive; therefore, DPSCs can be a promising cell source for regenerative medicine. The BMSCs and ADSCs show an age-dependent decline in their proliferative ability and regenerating capacity [6,21,22]. In fact, the volume of pulp tissue decreases with age, owing to secondary dentin formation and root canal mineralization [14]. The present study demonstrated that the number of collected cells from the pulp tissue of elderly patients was lower than that collected from young patients, which agrees with previously published data [23]. Both the quality and quantity of DPSCs are important in regenerative therapy; therefore, these age-related changes can make the clinical use of DPSCs from elderly patients challenging. However, the effect of age in bone regeneration has not yet been clear. Thus, age-independent culture methods that render the DPSCs to maintain a high proliferation and differentiation potential are desirable in tissue engineering therapy. Although some studies have reported an efficient system that induces osteogenic differentiation of DPSCs, such as producing the scaffold materials [24] and modifying the medium [25], its effect in aging patients has not yet been reported. We previously demonstrated that TH, a small osteogenic molecule, induced osteogenic differentiation of DPSCs from young patients in vivo and in vitro [15]. With this method, it is possible to culture DPSCs with a high proliferation and differentiation potential. In the present study, to assess whether TH can be used in DPSCs derived from elderly patients, we examined the effect of TH on the osteogenic differentiation potential of DPSCs from elderly patients in a mouse calvarial defect model and compared it with that of DPSCs from young patients.

It has been shown that MSCs from elderly patients have a lower proliferative potential even after the initial passage [8,26]. In the present study, we demonstrated that, at initial culture and P1, the proliferation potential of DPSCs from elderly patients was significantly lower than that of DPSCs from young patients. In contrast, no significant differences were found between the proliferative ability of elderly DPSCs and young DPSCs after P2, and the proliferative ability of elderly DPSCs was maintained at a high level even at P3. Moreover, elderly DPSCs demonstrated that the expression of NANOG and telomere length remained stable from P1 to P3 as well as young DPSCs, indicating that once the pulp cells gain “stemness” after passaging, the high proliferation potential of DPSCs is independent of age, even though the initial number of collected cells is low in elderly patients. These findings suggested that cellular senescence of elderly DPSCs have not been shown; furthermore, elderly DPSCs might maintain functional characteristics of stemness in short-term culture. Similar results were obtained in a previous study in which the authors observed a high proliferative ability of DPSCs derived from different age groups including elderly patients, and the ability was also maintained at P2 in all age groups [27]. 

We previously reported that the optimal concentration of TH that is the most effective on osteogenic differentiation of DPSCs is approximately 10^−6^ M and that OM is necessary for osteogenic induction when DPSCs are cultured with TH [15,28]. In the present study, we used the same concentration of TH and the same culture method for both young and elderly DPSCs. Osteogenic differentiation of DPSCs is a complex process characterized by the expression of Runx2, the early osteogenic marker as well as the main transcription factor, and timely expressed genes, such as ALP and Colla1, followed by extracellular matrix mineralization [29,30,31]. Our data demonstrated that compared with OM alone, OM with TH increased—although not significantly—the expression levels of ColIa1, Alp, osteocalcin, and Runx2 in both young and elderly DPSCs. These results were supported by immunocytochemistory analysis in the present study. We compared the nuclear intensity of Runx2 (the main transcription factor of osteogenesis) immunofluorescent signals detected in TH-induced DPSCs with that in OM. Our findings showed that Runx2 nuclear localization in TH-induced DPSCs was upregulated significantly in both young and elderly, indicating TH can regulate early osteogenic differentiation. Moreover, ALP staining and alizarin red S staining showed that TH induced higher ALP activity and more mineral deposition in both young and elderly groups, indicating TH is effective in inducing osteogenic differentiation of not only young DPSCs but also elderly DPSCs. 

As we previously reported, the cell sheet method using TH-induced DPSCs enables easier and safer transplantation of DPSCs [15]. Bone regenerative therapy using transplantation of DPSC sheets in combination with TH requires no scaffolds or growth factors, thus overcoming challenges associated with invasive autologous bone harvesting and ethical issues associated with allogenic bone transplantation and recombinant protein use. Moreover, previous studies have shown that DPSCs have a higher immunosuppressive activity and ability to prevent T cell alloreactivity in comparison with BMSCs [32,33], which can be advantageous for the clinical use of DPSC sheets. Furthermore, this finding was supported by the subsequent in vivo study using a mouse calvarial defect model. We evaluated the osteogenic potential of TH-induced DPSCs from elderly patients using a mouse calvarial defect model, which closely resembled clinical settings. Notably, transplants with elderly DPSCs and young DPSCs showed similar new bone formation at the edge of the calvarial defect. Moreover, BV and BMC in the transplants with elderly DPSCs were considerably higher than those in the control, but no significant difference was found between transplants with elderly and young DPSCs, indicating that elderly DPSCs treated with TH have a high potential for osteogenic differentiation and osteogenesis, which is not inferior to that of young DPSCs. Previous studies have shown that using scaffolds increased the osteogenesis of DPSCs [34,35] derived from young patients. However, TH improved the osteogenesis of DPSCs without any scaffolds and independent of the age of the source tissue. In conclusion, these radiological findings confirm the advantage of using TH for bone regenerative therapy for patients, irrespective of patient age.

There are also some limitations to this study. We compared young and elderly DPSCs using the cells in short-term culture from P0 to P3 in particular in this study. However, to detect the change of “aging”, we require studies using the cells in long-term culture. Furthermore, how the expression of osteogenic markers would be after transplantation of young and elderly DPSC sheets into mice remains unknown. Further research is thus needed. 

## 4. Materials and Methods 

### 4.1. Isolation of Dental Pulp Stem Cells

Human dental pulp stem cells were isolated from extracted teeth from 4 young (16–18 years old) and 4 elderly (41–54 years old) patients in good health (hereafter referred to as “young DPSCs” and “elderly DPSCs”, respectively)—after obtaining informed consent—at Tokyo Medical University Hospital. This study was approved by the institutional ethics committee of the Faculty of Medicine, Tokyo Medical University, Japan (approval No. 3486, approved on 4 October 2016). Dental pulp was extracted and minced into small pieces, followed by enzymatic digestion using 3 mg/mL collagenase type I (Sigma-Aldrich, St Louis, MO, USA) for 45 min at 37 °C; single-cell suspensions were obtained by passing the cells through a 70 µm cell strainer. The isolated cells were plated at a density of 1 × 10^5^ cells on 100 mm dishes and cultured in alpha-modified Eagle’s medium (αMEM; Gibco/BRL) supplemented with 15% fetal bovine serum (FBS; Biowest, Nuaillé, France) and 1% penicillin/streptomycin (P/S; Wako Pure Chemical Industries, Osaka, Japan). Cells were passaged at 70% confluence. Young and elderly DPSCs in passage 3 (P3) or P4 were used for experiments. Dental pulp stem cells were cultured in 12-well plates containing osteogenic differentiation media with and without TH. We used αMEM supplemented with 10% FBS, 1% P/S, and 10 nM dexamethasone (Wako Pure Chemical Industries) for osteogenic induction (osteogenic medium (OM)). We added TH (Takeda Chemical Industries, Osaka, Japan) at the optimal concentration of 10^−6^ M to the OM, as described previously [15].

### 4.2. Cell Proliferation

Dental pulp stem cells were cultured in 100 mm dishes at a density of 1 × 10^5^ cells (P0) until they reached 70% confluence with regular medium (αMEM, 15% FBS, 1% P/S). To calculate the initial percentage of DPSCs from young and elderly patients, we counted the number of cells initially plated and the number of cells after detachment at 70% confluence using a hemocytometer. When counting the number of cells, we performed trypan blue staining in order to count only the live cells that were unstained in blue. Cell proliferation of young and elderly DPSCs was compared by collecting and counting cells at P1, P2, and P3. Cell Counting Kit-8 (Dojindo, Kumamoto, Japan) was also used to analyze the proliferation rate. Young and elderly cells at P2 were seeded at 1 × 10^3^ cells per 96-well plate. The labeling mixture was added, and cells were incubated for 2 h in a 37 °C/5 % CO_2_ incubator. Cell numbers were measured using a spectrophotometer at 450 nm absorbance at 24, 48, and 72 h of culture.

### 4.3. Telomere Length

Genomic DNA from young and elderly DPSCs at P1 and P3 was extracted using PureLink Genomic DNA mini kit (Invitrogen, Carlsbad, CA, USA) according to the manufacturer’s instructions. Telomere length was analyzed by using Absolute Human Telomere Length Quantification qPCR Assay Kit (ScienCell Research Laboratories, San Diego, CA, USA), following the manufacture’s protocol. PCR products were analyzed in 3% agarose gel electrophoresis at 100 V for 90 min. Bands were visualized by UV light.

### 4.4. Flow Cytometry Analysis

A single-cell suspension was obtained by detaching the cells using 0.25% trypsin and washing them with phosphate-buffered saline (PBS). Cells were fixed with 10% FBS for 10 min at 37 ℃ and then incubated with the following antibodies for 90 min at 4 ℃: phycoerythrin (PE)-conjugated human CD14, PE-conjugated human CD29, PE-conjugated human CD34, PE-conjugated human CD44, PE-conjugated human CD73, PE-conjugated human CD81, PE-conjugated human CD90, and PE-conjugated human CD105 (BioLegend, San Diego, CA, USA). Cells were washed with PBS and then fixed in 4% paraformaldehyde (PFA) for 10 min at 4 ℃. Each isotype controls were used. Mouse IgG1κ (BioLegend) was used for CD29, CD44, CD73, CD81, CD90, and CD105. Mouse IgG2aκ (BioLegend) was used for CD14 and CD34. The antibody dilutions and clone number used in this study are listed in Table S1. Cells were analyzed using a flow cytometer (BD Biosciences, San Jose, CA, USA), and data analysis was performed using FlowJo software (FlowJo; FlowJo, LLC, Ashland, OR, USA).

### 4.5. Osteogenic Differentiation

Dental pulp stem cells were cultured in 12-well plates containing osteogenic differentiation media with and without TH for 14 days. We used αMEM supplemented with 10% FBS, 1% P/S, and 10 nM dexamethasone (Wako Pure Chemical Industries) for osteogenic induction (osteogenic medium (OM)). We added TH (Takeda Chemical Industries) at the optimal concentration of 10^−6^ M to the OM, as described previously [15].

### 4.6. Alizarin Red S Staining

Young and elderly DPSCs were cultured in OM with or without TH for 14 days and stained with alizarin red S (Sigma-Aldrich), as described previously. In brief, cells were fixed with 10% formaldehyde in PBS for 10 min at 4 ℃, followed by two washes with distilled water. Next, the cells were stained in 1% alizarin red S solution for 15 min. After staining, they were washed twice with distilled water. Quantification of relative staining intensities was calculated by ImageJ software (National Institutes of Health, Bethesda, MD, USA).

### 4.7. Alkaline Phosphatase Staining

Young and elderly DPSCs were stained with alkaline phosphatase (ALP), as described previously. In brief, the cells were fixed in 70% ethanol after they were rinsed with PBS, followed by staining for 10 min with 0.01% naphthol AS-MX phosphate (Sigma-Aldrich), using 1% N,N-dimethyl formamide (Wako Pure Chemical Industries) as the substrate and 0.06% Fast BB salt (Sigma-Aldrich) as a coupler. Quantification of relative staining intensities was calculated by ImageJ 1.40 g freeware.

### 4.8. Immunocytochemistry

Immunocytochemistry was performed in young and elderly DPSCs cultured with OM and OM with TH. After 7 days of incubation, cells were washed with PBS and fixed with 4% paraformaldehyde for 30 min. Before we applied the antibody, cells were blocked with 10% normal goat serum (NGS) in PBS with 0.1% Tween-20 (PBT) for 1 h. After blocking, cells were incubated overnight at 4 ℃ with primary antibody against Runx2 (1:1000; Abcam, Cambridge, UK; ab192256) and visualized using goat anti-human IgG Alexa Fluor 594 (1:300; Molecular Probes) for 1 h at room temperature. Hoechst 33342 dye (1:2000; Sigma-Aldrich) was added for nuclear staining. Fluorescence images were captured using a Zeiss LSM 700 confocal microscope using ×40 oil immersion objective (laser line, 543 nm; average pixel time, 4 μs). Integrated optical density was analyzed using the method described previously by Tamás et al. [36]. Briefly, integrated density of nuclei of 30 independent cells in randomly selected field of view was measured using ImageJ software. We compared the nuclear intensity of Runx2 immunofluorescent signals detected in cells that treated with TH to cells that were treated with OM.

### 4.9. Reverse Transcription Polymerase Chain Reaction Analysis

Total RNA from young and elderly DPSCs cultured in regular medium (RM Dulbecco’s modified Eagle’s medium (DMEM; GIBCO/BRL) supplemented with 10% FBS and 1% P/S), OM, and OM with TH was isolated using TRIzol (Invitrogen), and reverse transcription was performed using a QuantiTect Reverse Transcription kit (Qiagen), according to the manufacturer’s instructions. Real-time polymerase chain reaction of *NANOG*, stem cell marker was performed using young and elderly DPSCs cultured in RM. Furthermore, *Runx2*, an osteoprogenitor maker; *Alp* and *ColIa1*, early markers of osteoblast differentiation; and *osteocalcin*, a mature osteoblast marker, was performed with OM and OM with TH-induced DPSCs in a Lightcycler 96 (Roche Diagnostics, Basel Switzerland) using THUNDERBIRD SYBR qPCR Mix (Toyobo, Osaka, Japan), as described previously. *GAPDH* was used as an endogenous control. The primer sequences used in this study are presented in Table 2. The 2^−ΔΔCT^ method was used to calculate relative expression levels.

### 4.10. Transplantation of Young and Elderly Dental Pulp Stem Cell Sheets into a Mouse Calvarial Defect Model

A mouse calvarial defect model was used for animal experiments, following the guidelines of the Animal Care and Use committee of the Faculty of Medicine, Tokyo Medical University (approval No. R2-0063). We used 7-week-old male NOD.CB17-Prkdc^scid^/J (NOD SCID) mice (Oriental Yeast, Tokyo, Japan). The mice were anesthetized with medetomidine (0.75 mg/kg), midazolam (4.0 mg/kg), and butorphanol tartrate (5.0 mg/mL) via intraperitoneal injection. The calvaria were exposed after skin and muscle layer incision. Defects with a diameter of 3.5 mm were created using biopsy punches (Kai Corporation, Tokyo, Japan) in the right parietal bone. Young and elderly DPSCs were cultured on 12-well temperature-responsive dishes (Upcell, CellSeed) at a density of 1 × 10^4^ cells in OM with TH treatment for 14 days. To harvest cell sheets, we placed dishes at 20 °C for 30 min. Each sheet was transplanted into the calvarial defects carefully. Skin was closed with 6–0 nylon sutures. Four mice were used in each experimental group, consisting of the young DPSCs, elderly DPSCs, and control. For the control group, we only created defects, and nothing was transplanted. Eight weeks after transplantation, we sacrificed the mice and harvested the calvaria.

### 4.11. Radiological Evaluation

The calvarial defects were examined by micro-computed tomography (micro-CT; SMX-90CT, Shimadzu, Kyoto, Japan) The scanning conditions were as follows: 90 kV, 110 µA, and a field of view (XY) of 10 mm; the resolution of one CT slice was 512 × 512 pixels. The data were reconstructed and analyzed using the morphometric software TRI/3D-BON (Ratoc System Engineering, Tokyo, Japan). Bone volume (BV), bone mineral content (BMC), and bone mineral density (BMD) of a 5 × 5 × 3 mm^3^ cuboid area located in the center of the initial defect area were calculated.

### 4.12. Statistical Analysis

Each experiment was replicated at least three times. Statistical analysis was performed using SPSS 24.0 software (IBM, Armonk, NY, USA); all data are expressed as mean ± SD. Statistical significance was evaluated using one-way ANOVA, and *p*-values were calculated with Student’s *t*-test. *p* < 0.05 or *p* < 0.01 were considered indicative of statistically significant differences between two groups.

## 5. Conclusions

Our study demonstrated that TH is effective in inducing osteogenic differentiation of DPSCs from elderly patients. Moreover, TH-induced DPSC from elderly patients have a high potential for osteogenesis as well as young patients in vivo, indicating that TH-induced DPSCs from elderly patients can be a useful cell source for bone regenerative medicine. 

## Figures and Tables

**Table 1:** Initial percentage of dental pulp stem cells (DPSCs) derived from young and elderly patients.

	Number of Collected Cells (×10^4^)	Number of DPSCs at 70 % Confluence (×10^4^)	Percentage of DPSCs (%)	Average Percentage of DPSCs (%)
Young				
Y1 (18 year)	1000	800	80	56
Y2 (16 year)	1000	500	50	
Y3 (18 year)	2600	600	23	
Y4 (17 year)	670	480	71	
Elderly				
E1 (41 year)	198	107	54	31
E2 (54 year)	60	10	16	
E3 (50 year)	760	60	7	
E4 (42 year)	63	28	44	

**Table 2:** Sequence information of primers used for quantitative real-time PCR.

Gene	Primer Sequences (Forward and Reverse, 5′-3′)	Accession
*GAPDH*	GAAGGTGAAGGTCGGAGTCA GAAGATGGTGATGGGATTTC	BC023632
*NANOG*	AACTGGCCGAAGAATAGCAA TGCACCAGGTCTGAGTGTTC	NM_024865
*RUNX2*	CAGACCAGCAGCACTCCATA CAGCGTCAACACCATCATTC	NM_004348
*ALP*	ATGAAGGAAAAGCCAAGCAG ATGGAGACATTCTCTCGTTC	NM_000478
*COLIA1*	GTGCTAAAGGTGCCAATGGT CTCCTCGCTTTCCTTCCTCT	NM_000088
*Osteocalcin*	GGCAGCGAGGTAGTGAAGAG AGCAGAGCGACACCCTAGAC	NM_199173

## Supplementary Materials

The following are available online at https://www.mdpi.com/1422-0067/21/20/7731/s1, Table S1: Antibody dilutions and clone numbers for the FACS analysis.

## Abbreviations

ADSCs	adipose tissue-derived stem cells
ALP	alkaline phosphatase
BMC	bone mineral content
BMD	bone mineral density
BMP	bone morphogenic protein
BMSCs	bone marrow stem cells
BV	bone volume
COLIA1	type I collagen alpha 1
DPSCs	human dental pulp stem cells
FITC	fluorescein isothiocyanate
MSCs	mesenchymal stem cells
OM	osteogenic medium
P/S	penicillin/streptomycin
PBS	phosphate-buffered saline
PE	phycoerythrin
RM	regular medium
SD	standard deviation
TH	4-(4-methoxyphenyl) pyrido[4 0,30:4,5]thieno[2,3-b]pyridine-2-carboxamide
αMEM	alpha-modified Eagle’s medium
