# Supplementary material for: Bone Regeneration Potential of Human Dental Pulp Stem Cells Derived from Elderly Patients and Osteo-Induced by a Helioxanthin Derivative
Source: Int J Mol Sci. 2020 Oct 19;21(20):7731. doi: 10.3390/ijms21207731 (PMC7590053; doi:10.3390/ijms21207731)
Supplement: Supplementary file 1 [file ijms-21-07731-s001.pdf]

Table S1. Antibody dilutions and clone numbers for the FACS analysis

Antibody	Clone number	Dilution
PE anti-human CD14	M5E2	1:20
PE anti–human CD29	TS2/16	1:20
PE anti–human CD34	561	1:20
PE anti–human CD44	BJ18	1:20
PE anti-human CD73	AD2	1:20
PE anti-human CD81	5A6	1:20
PE anti-human CD90	5E10	1:20
PE anti-human CD105	43A3	1:20
